# Beyond the Visual Word Form Area – a cognitive characterization of the left ventral occipitotemporal cortex

**DOI:** 10.3389/fnhum.2023.1199366

**Published:** 2023-07-28

**Authors:** Agnieszka Dȩbska, Marta Wójcik, Katarzyna Chyl, Gabriela Dziȩgiel-Fivet, Katarzyna Jednoróg

**Affiliations:** ^1^Laboratory of Language Neurobiology, Nencki Institute of Experimental Biology, Polish Academy of Sciences, Warsaw, Poland; ^2^The Educational Research Institute, Warsaw, Poland

**Keywords:** Visual Word Form Area, left ventral occipitotemporal cortex, orthography, phonology, reading, language

## Abstract

The left ventral occipitotemporal cortex has been traditionally viewed as a pathway for visual object recognition including written letters and words. Its crucial role in reading was strengthened by the studies on the functionally localized “Visual Word Form Area” responsible for processing word-like information. However, in the past 20 years, empirical studies have challenged the assumptions of this brain region as processing exclusively visual or even orthographic stimuli. In this review, we aimed to present the development of understanding of the left ventral occipitotemporal cortex from the visually based letter area to the modality-independent symbolic language related region. We discuss theoretical and empirical research that includes orthographic, phonological, and semantic properties of language. Existing results showed that involvement of the left ventral occipitotemporal cortex is not limited to unimodal activity but also includes multimodal processes. The idea of the integrative nature of this region is supported by the broad functional and structural connectivity with language-related and attentional brain networks. We conclude that although the function of the area is not yet fully understood in human cognition, its role goes beyond visual word form processing. The left ventral occipitotemporal cortex seems to be crucial for combining higher-level language information with abstract forms that convey meaning independently of modality.

## 1. Introduction

The unprecedented interest of neuroscientists in the structure and function of the left ventral occipitotemporal cortex (VOT) came from the fact that this region has over the years been systematically reported as particularly responsive during processing of written words (McCandliss et al., 2003; Dehaene and Cohen, 2011). Typically language-related left VOT is anatomically situated within the left occipitotemporal sulcus extending to the left fusiform gyrus and inferior temporal gyrus (Price and Devlin, 2011; Price, 2012; Lerma-Usabiaga et al., 2018).

**FIGURE 1:**
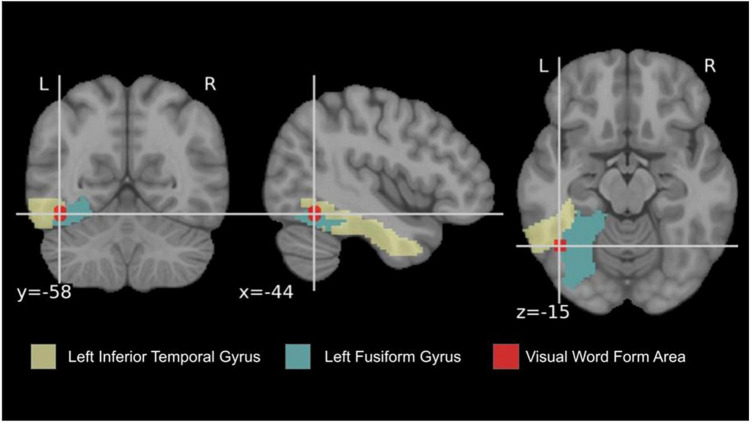
Anatomical visualization of the left inferior temporal gyrus and fusiform gyrus; the localization of the word-specific peak from the meta-analysis of Jobard et al. (2003).

The term Visual Word Form Area (VWFA) – functionally localized in most studies in the mid-anterior part of the left VOT – was mostly popularized by Cohen et al. (2000, 2002). The most canonical proposition of the VWFA localization given by Cohen et al. (2002) was: *x* = −42, *y* = −57, *z* = −15. Later the meta-analysis by Jobard et al. (2003) of language-based contrasts (e.g., word-fixation and word-consonants) defined the localization at: *x* = −44, *y* = −58, *z* = −15 (see: Figure 1). However, the exact localization of the peak is assumed to slightly differ in each individual and was typically tested by comparing activation of the left mid-fusiform for real words versus pseudowords, consonant strings, checkerboards, false fonts, symbol strings, or other letter-like visual stimuli. There is an ongoing debate if the VWFA as a part of higher-level visual cortex should show preferential activation to visual words over other categories of namable visual stimuli such as objects, tools, or faces (see e.g., Price and Devlin, 2003; Szwed et al., 2011; Price, 2012; Nestor et al., 2013; Neudorf et al., 2022). Hirshorn et al. (2016) investigated the visual word form hypothesis using direct brain stimulation and EEG. They focused on the left midfusiform gyrus (lmFG) and discovered that disrupting activity in this region impaired perception of words and letters. The lmFG is involved in different stages of orthographic representation, including low-level visual representation.

In the critical article Price and Devlin (2003) showed that the VWFA (*x* = −42, *y* = −57, *z* = −15) is involved not only in visual word processing but also in naming colors and objects, reading Braille and processing auditory words. This was one of the first pieces of evidence that the middle left VOT area called VWFA is involved in more than only processing visual word forms. What is interesting is that all those tasks were connected to language functions, such as naming (semantics), speech sound processing (phonology), and Braille reading (orthography but in a modality different than visual). Therefore, in the current article, we focus on the language-specific activity of the left-lateralized VOT (LS-VOT) in multiple language-related tasks. We discuss phonological, semantic, and orthographic processing within the LS-VOT independently from visual, auditory, or tactile modality. We start by describing the earliest but also the narrowest understanding of the LS-VOT as a visual-based reading region and extend this by adding different layers of evidence for other possible language-related functions of this area.

## 2. LS-VOT as a part of the visual letter-related system

The cognitive concept of the Visual Word Form came originally from the Warrington and Shallice (1980) clinical study where authors contrasted two different forms of reading: letter-by-letter and whole words. The ability to extract an abstract orthographic representation of a whole word, irrespective of its color, shape, font, and size constitutes the knowledge of the “visual word form.” According to Rapp and Fischer-Baum (2014), orthographic representations may vary in the size of units (morphemes, digraphs, and letters) and should contain information about letter identity, consonant-vowel status, syllabic role, and letter position.

From the beginning, the chosen terminology defined the scope of the investigation as modality-dependent since a VISUAL Word Form concept drove authors to explore the VWFA as a part of the human visual system. The first studies on the VWFA focused on how reading acquisition transforms the “inferotemporal pathway for visual object recognition” (McCandliss et al., 2003). The visual modality-based approach proposed that LS-VOT is tuned to processing letters based on the constraints in the hierarchical receptive field structure (Dehaene et al., 2005) with a preference for line junctions (Szwed et al., 2011). Accordingly, internal segmentation of the LS-VOT is functionality dependent on the level of word-related form processing. A posterior-to-anterior gradient in selectivity was revealed, where false fonts, letters, bigrams, trigrams, and whole words are processed with increased selectivity in a posterior-to-anterior direction (Vinckier et al., 2007). This gradient is consistent with Dehaene et al.’s (2005) proposal that the left posterior VOT processes single letters, the middle processes bigrams and trigrams, and the anterior processes lexical units. Studies that combine relatively high spatial and temporal resolutions confirm that when showed that letter-selective responses occurred approximately 60 ms earlier than word-selective responses, with phase-locking in anterior areas involved in lexico-semantic processing (Thesen et al., 2012). In this visual-based approach, LS-VOT is treated more as an identifier for visual properties of letters and word-forms based on line junctions than as a part of the language-specific system. However, this perspective poses some problems.

First, it neglects that LS-VOT is structurally (Saygin et al., 2016; Vanderauwera et al., 2018; Moulton et al., 2019) and functionally (Morken et al., 2017; Smith et al., 2018; Li et al., 2020) connected with the language system and especially the left superior temporal gyrus (STG) and inferior frontal gyrus (IFG) which take part in phonological and semantic processing (Hodgson et al., 2021). It seems that not only does shape, size or font determine orthographic form but also its phonology (Pylkkänen and Okano, 2010). In terms of LS-VOT engagement, Hashimoto and Sakai (2004) showed preferential activation to newly learned orthographic forms in adults but only when they were combined with speech sounds instead of non-speech sounds (tones and noise).

Second, orthographic representations are usually understood as modality-independent (Rapp and Fischer-Baum, 2014). In line, the LS-VOT was found to be responsive to reading not only in the visual but also in the tactile modality (Büchel et al., 1998a,b). This was true for both early and congenitally blind individuals (Amedi et al., 2003), some late blind individuals (Burton et al., 2002), as well as sighted individuals learning to read Braille tactually (Siuda-Krzywicka et al., 2016). The site of the reading-related activation was not discernable between the blind and the sighted readers (Reich et al., 2011; Dziȩgiel-Fivet et al., 2021) and in both groups, similar sensitivity to letters was found (Ra̧czy et al., 2019). These observations led to a hypothesis that LS-VOT is implicated in reading on a modality-independent level (Amedi et al., 2017).

Third, LS-VOT activation may not be specific to letter shapes as compared to other visual meaningful stimuli like nameable pictures. Neudorf et al. (2022) showed that the same LS-VOT area was equally responsive to naming pictures and words without dominant activations for one type of stimuli. There is also an interesting line of research that broadens the LS-VOT function to all symbolic stimuli with known referents, even those without an orthographic structure or preferred visual properties like line junctions. In a study by Song et al. (2012) the LS-VOT was more active for symbolic landscapes (e.g., Eiffel Tower as a symbol of Paris) than for non-symbolic landscapes (e.g., unknown high building). This effect was also present for the artificial symbol–referent associations that were trained in the study. Preferential LS-VOT activity was further found for stimuli with communicative intentions like body gestures (Xu et al., 2009), or visually complex everyday scenes (Tylén et al., 2009). A more general view was proposed by Vingerhoets et al. (2013) who showed mutual co-lateralization between tool use and language, regardless of handedness. The authors argue that their results link gestures and speech to explain the beginning of human language.

## 3. LS-VOT as storage for abstract orthographic representations

The idea that the LS-VOT is storage for abstract orthographic forms was widely explored. There is an ongoing debate about whether LS-VOT processes whole-word (lexical) forms or whether LS-VOT is sensitive primarily to smaller orthographic parts like letters or bigrams (Dehaene et al., 2005; Binder et al., 2006). There was also a more hybrid approach claiming that LS-VOT is involved in both lexical and sublexical coding (e.g., Schurz et al., 2010). The sublexical hypothesis which assumed that the LS-VOT is responsive to bigrams or trigrams rather than whole words was challenged by Glezer et al. (2009). The authors employed a rapid adaptation paradigm to detect more subtle changes in BOLD signal than in the usual contrast testing. With this change in methodology, the authors claimed that LS-VOT proved to be selective to real words and not sublexical units. In their study, the comparison of mean activations for two real words that shared visual similarities but were not identical (like “farm” – “form”) revealed no neural adaptation patterns contrary to the presentation of two orthographically identical words (Glezer et al., 2009). Thus, sublexical information shared by those words was not sufficient enough to evoke selectivity in LS-VOT. Further research revealed that LS-VOT’s selectivity was also present in processing familiarized (trained) pseudowords in contrast to the novel ones (Glezer et al., 2015). This approach corresponds with another type of research based on the so-called “familiarity effect.” It was first observed in object recognition studies when the repeated presentation of the stimuli led to decreased activation of the VOT (Chao et al., 2002; Van Turennout et al., 2003). Following this idea, Kronbichler et al. (2004) tested the LS-VOT response regarding the stimuli arranged gradually from the most familiar (high-frequency words) to the least (pseudowords). Again, the authors demonstrated that the higher the familiarity, the lower the LS-VOT activation, which was interpreted as a proof for the whole-word functionality instead of letter-based functionality: more effort was needed to decode words that did not have familiar forms. In the follow-up study in 2007, Kronbichler et al. (2007) used a different set of items (words/pseudohomophones/pseudowords) and observed the same effect: lower activations of LS-VOT for words versus pseudohomophones and pseudowords [this was widely replicated by Bruno et al. (2008), Woollams et al. (2011), and Wimmer and Ludersdorfer (2018)]. The latest connectivity studies (Lerma-Usabiaga et al., 2018; White et al., 2019; Yablonski et al., 2023) showed that anterior LS-VOT is connected with higher-language areas as opposed to the posterior part connected with visual regions and sensitive to visual features of words. It may suggest the functional division between posterior and anterior parts, where anterior part showed growing multimodality and holistic lexical processing while a posterior part supports this hub by providing information about visual features of letters.

There is a consensus that especially the location of left-hemispheric LS-VOT is very consistent across tasks and writing systems (Bolger et al., 2005; meta-analysis including studies carried out in European alphabets, Chinese characters, and Japanese Kana and Kanji). In children, the overlap of specific activation for reading English words and Chinese characters (Krafnick et al., 2016), as well as French words and Chinese characters (Feng et al., 2021) was found in the left LS-VOT in the direct proximity of the English-based ROI (Cohen et al., 2002). Classical LS-VOT ROI was also explored in adults reading frequent Chinese characters (nouns), and activation in this region was confirmed as specific for reading (Liu et al., 2008). No language differences and high specificity of the LS-VOT [also located as in Cohen et al. (2002)] were also found in bilinguals reading Chinese and Korean (Bai et al., 2011). Nonetheless, while the LS-VOT engagement was consistent across many studies regardless of the languages and writing systems, its right hemisphere homolog was consistently found to be more engaged in the readers of logographic compared to alphabetic scripts (e.g., Bolger et al., 2005). The bilateral processing of non-alphabetic scripts has been attributed to their visual complexity and the larger grain size principle inherent in these scripts (Hsiao and Lam, 2013); right hemisphere engagement would be thus more associated with holistic or whole-object processing (Hirshorn and Harris, 2022). In line with that Li et al. (2017) showed that, similarly to alphabetic languages, in readers of logographic scripts the connection between spoken language areas and the LS-VOT strengthens as individuals grow older. However, the strength of this connectivity differs between the orthographies, as learning alphabetic languages is mostly based on phonics instructions.

## 4. Why is LS-VOT sensitive to speech?

Language Specific - VOT (LS-VOT) was traditionally argued to store visual-based orthographic representations. However, many studies so far showed that the LS-VOT is activated in auditory speech-based tasks. This was found in sighted readers (Dehaene et al., 2015; Planton et al., 2019) but also to a larger extent in blind Braille readers. In the case of the blind population, VOT activation was observed during both low-level tasks like passive listening to words (Kim et al., 2017; Dziȩgiel-Fivet et al., 2021), basic phonological tasks (Burton et al., 2003; Arnaud et al., 2013) but also during high-level tasks like sentence comprehension where the activity of this region was related to task difficulty (Kim et al., 2017).

Moreover, in the sighted LS-VOT’s activation seems to be related to the overall reading level and reading deficits (Dehaene et al., 2010; Desroches et al., 2010; Dehaene and Cohen, 2011; Dȩbska et al., 2016, 2019; Wang J. et al., 2018, Wang et al., 2021). This is in analogy to the theories of reading which suggest that the involuntary orthographic influence in processing phonology appears as a function of reading skill (Morais et al., 1979; Ventura et al., 2007; Ziegler and Muneaux, 2007). The plethora of empirical evidence favors the abstract, modality-independent concept of the LS-VOT functionality. So far several hypotheses have been formulated that describes an extended LS-VOT role in speech processing.

### 4.1. A unimodal orthographic hypothesis

Dehaene et al. (2010) and Dehaene and Cohen (2011) proposed a unimodal orthographic hypothesis. Accordingly, abstract orthographic representations are activated automatically in a top-down manner during active speech processing. In this view VOT activations are not a result of phonological processing but involuntary orthographic co-processing that facilitates and accompanies phonological processing. In Dehaene et al. (2010) study, LS-VOT was active during auditory lexical decision task (distinguishing between words and pseudowords) but not in passive sentence listening. The activity in the auditory lexical decision task was present only in literates but not in illiterates. Individual differences in the strength of LS-VOT activity on auditory stimuli correlated with the LS-VOT activity for written words but not other visual objects. These empirical findings were interpreted as evidence for the unimodal orthographic hypothesis. Ludersdorfer et al. (2015, 2016) asked whether the need for orthographic processing is crucial for LS-VOT’s activation in an auditory speech-based task. Results showed that LS-VOT was more active for orthographic (letter count) relative to semantic (living-non-living) tasks (both were based on speech stimuli). However, even the semantic task elicited weak activation relative to the baseline non-linguistic tones processing in the LS-VOT. Most auditory speech-based tasks where the preferential activity of LS-VOT and its positive relation to reading proficiency was acknowledged were not orthographic tasks *per se*. Most empirical data came from phonological awareness tasks like word rhyming (Booth et al., 2002; Desroches et al., 2010; Yoncheva et al., 2010; Dȩbska et al., 2016), alliteration (Wang J. et al., 2018; Dȩbska et al., 2019; Wang et al., 2021), or even pseudoword matching (Dȩbska et al., 2019). The question arises if the preferential activity of LS-VOT in speech-based tasks requires the active engagement of orthographic code [like Dehaene et al. (2010) directly proposed], or if LS-VOT is crucial for processing phonological or semantic properties of language independent of the need to activate orthographic code (see sections “2. LS-VOT as a part of the visual letter-related system” and “4.2. A unimodal phonological hypothesis”).

Another issue is that the LS-VOT might play a different role in the speech-based task than in processing written stimuli. For example, Ludersdorfer et al. (2013) showed that in the case of visual stimuli, the LS-VOT reduced its activity for word-like written stimuli (words < pseudowords < artificial stimuli) but in reverse increased its activity for word-like auditory stimuli (words and pseudowords > artificial stimuli). Also, observed LS-VOT engagement during the processing of speech might be related to the deactivation effects more than activation differences. For example, Yoncheva et al. (2010) controlled the attentional demands of two tasks: one speech-based and one non-linguistic (tones comparison). The comparison between attentional tone and speech processing showed that both conditions evoked deactivations in the left fusiform except for the more narrow left mid-fusiform area in the case of a speech condition. This leads to higher activity in the mid-fusiform (corresponding to VWFA) relative to surrounding deactivated regions of the left fusiform.

### 4.2. A unimodal phonological hypothesis

A second explanation of the LS-VOT’s preferential activation to speech is the existence of neuronal populations involved in processing phonology. Pattamadilok et al. (2019) formulated a theoretical proposal for the existence of unimodal (auditory) phonological groups of neurons in LS-VOT that are sensitive to phonological but not orthographic features of stimuli. The preliminary confirming evidence came from the Conant et al. (2020) studies on school-aged children and adolescents. They found that activity in the LS-VOT was modulated by phonological factors such as syllable structure, suggesting that this region is involved in processing the sound structure of language. The authors found that the activity of LS-VOT in discriminating speech-syllables versus non-phonemic stimuli was not only positively related to the reading level but also to categorical phoneme perception skills. The authors concluded that LS-VOT plays a role in both reading proficiency and phoneme perception refinement. Although the authors did not exclude an automatic orthographic co-activation explanation, they rather argued that LS-VOT is responsible for the refinement of speech perception and audiovisual integration (see section “4.3. A multimodality hypothesis”).

Moreover, Xue et al. (2006) trained adult individuals with a new visual font for 2 weeks (visual training) and then new sounds for another 2 weeks (phonological training). Interestingly, phonological training evoked more activity in bilateral LS-VOT compared to visual training which made the authors conclude that (bilateral) LS-VOT is engaged in processing phonology or in phonology-orthography integration. Still, the interpretation of results was limited by the fact that the order of training was not counterbalanced.

Another piece of evidence for the phonological function of LS-VOT was brought by Pattamadilok et al. (2009) in a TMS facilitation study. The authors tested whether in orthographic or phonological priming on LS-VOT, a TMS stimulation would facilitate word processing. Results showed only unimodal (orthographic or phonological) but not multimodal facilitation effects. The authors interpreted those findings as a confirmation on the existence of separate phonological and orthographic groups of neurons within the LS-VOT but not multimodal ones. However, the site of the LS-VOT stimulation was chosen based on non-specific individual localizer (words > fixation) therefore the localization of phonological versus orthographic LS-VOT areas is still a matter of debate and probably requires higher spatial resolutions than the ones offered by standard fMRI or TMS (Grill-Spector and Malach, 2001; Price and Devlin, 2003).

### 4.3. A multimodality hypothesis

One may ask if the positive relationship between orthography-phonology integration in the LS-VOT and the reading level (e.g., Wang J. et al., 2018), may be due to the fact that LS-VOT integrates information from orthography and phonology. Print-speech convergence and audiovisual integration effects in LS-VOT that were found to be reading-related are worth discussing in this context.

First, in the case of print-speech convergence, there is an assumption that literacy acquisition changes the brain in a way that print stimuli start to be processed in the network responsible for spoken language processing (Liberman, 1992; Rueckl et al., 2015; Preston et al., 2016; Chyl et al., 2018). Most of the studies showed print-speech coactivations in relation to the reading level mostly in the left IFG and STG (Frost et al., 2009; Rueckl et al., 2015; Preston et al., 2016; Chyl et al., 2018; Marks et al., 2019): regions typically involved in speech sound processing. Interestingly the LS-VOT area also showed print-speech convergence in the opaque orthographies like English or Hebrew compared to the more transparent orthographies like Spanish (Rueckl et al., 2015). This effect was evident in English adults (Rueckl et al., 2015) and beginning 7 years old readers (Chyl et al., 2021). However in Chyl et al. (2021) no evidence was found on the relation between print-speech co-activation in LS-VOT and the reading level in English or Polish (see Chyl et al., 2021, Supplementary Material 4). Also, the representational similarity analysis (RSA) in processing print and speech in beginning readers showed no significant overlap in LS-VOT contrary to the left IFG and STG. Different results were found in the case of young Chinese adults (18–26 years olds) where RSA analysis showed that LS-VOT patterns of activation are best explained by orthographic and phonological similarity compared to unimodal processing (Zhao et al., 2017; Qu et al., 2022). So it seems that similar patterns between phonology and orthography might be related to reading ability, yet more cross-linguistic and developmental studies are needed.

Second, in the case of language-specific audiovisual integration, involvement in LS-VOT was found during audio-visual exposure to letters and ambiguous speech sounds (Romanovska et al., 2021). McNorgan and Booth (2015) tested audiovisual integration specifically in LS-VOT in English 8–13 years old children in relation to reading and found that multisensory integration in LS-VOT was modulated by the reading level.

### 4.4. An “all in one” hypothesis

The last possibility is that orthographic, phonological, semantic, and multimodal groups of neurons coexist within the LS-VOT. Those groups could be spatially separated and therefore it should be possible to identify their individual topography. Alternatively, the areas might not be spatially distinct for different functions and thus the activation patterns measured by multivoxel pattern analysis of the same or similar area will differ depending on the task. These two alternatives are interesting because at the first sight the “all in one” approach allows the reconciliation of different empirical findings. An interesting example of the “all in one” approach is an “impostor” hypothesis proposed by Cohen et al. (2004). When the authors tested written and auditory word processing in VOT they found a multimodal area called LIMA (“lateral inferotemporal multimodal area”). LIMA was localized anteriorly to the canonical localization of VWFA (LIMA = *x* = −54, *y* = −52, *z* = −10, VWFA = *x* = −39, *y* = −60, *z* = −18, Talairach coordinates). During the presentation of written and auditory words LIMA was activated independently of modality whereas the region described as VWFA was active only in response to the visual words. The authors concluded that VWFA and LIMA might be “easily confounded” when interpreting the results of fMRI studies (see also Sebastian et al., 2014). Curiously, the authors gave LIMA a function that was later discussed in the context of VWFA: an integrative hub of cross-modal phonemic and lexical links (Price and Devlin, 2011). The “all-in-one” approach is promising because instead of focusing on VWFA defined as an area preferentially activated to real words, the focus is placed more on processing different functions like orthography or phonology or both those types of information within the VOT. The question is how those spatially different groups of neurons or functionally different patterns of activations should be localized on the individual level, analogically to how VWFA was localized. The promising approaches of using fMRI rapid adaptation (Weiner et al., 2010) or fast periodic stimulation (Gao et al., 2018) are yet to be developed. Interesting insights came from multivoxel pattern analysis research (MVPA). A univariate analysis, which averages brain activation across voxels, could fail to detect weak but informative signals distributed in multivariate patterns (Tong and Pratte, 2012). Different neural patterns are suggested to be associated with different cognitive functions, therefore, may distinguish between various roles of LS-VOT. Also, the MVPA might be more sensitive to individual differences in word processing and reading styles (Carlos et al., 2019). Fischer-Baum et al. (2017) showed that the same region of LS-VOT encodes orthographic and semantic (but not phonological) information. This semantic processing seems to be task- and category-dependent and may contribute to orthographic identification and overall task performance (Wang X. et al., 2018). Another promising approach was presented by Kay and Yeatman (2017). Their model for faces and words demonstrates that high-level representations in the cortex are built from and tied to low-level properties through linear and non-linear operations. This suggests that visual features hierarchically contribute to semantic representations. The model provides a potential mechanism for the emergence of semantic tuning properties in the visual cortex.

## 5. LS-VOT as a part of distributed neural systems

The research described so far was mostly based on the assumption of a localist representation system, which means that it assumes one set of neurons that is responsible for processing one concept. Instead, a distributive approach may be taken into account (e.g., Roy, 2017) where the whole collective brain network is responsible for encoding a concept. This is an interesting approach because it seems to reconcile different lines of research. For example, there is tension between the purely unimodal nature of LS-VOT and its multimodal characteristics. In the case of distributed system approach, we may treat the LS-VOT as a part of the multimodal network but the function within that network may be unimodal or may vary across tasks (e.g., see Price and Devlin, 2011). Also, the discussion on the LS-VOT’s sensitivity for active speech tasks rather than for passive listening, may be informed by the research on connections between LS-VOT and the attention network. If LS-VOT is a part of two cortical networks, the differences in the activity of LS-VOT in active speech tasks vs. passive listening may be a result of the interaction between roles the left VOT plays in language and attention networks. If this is the case, firstly, we may ask what is the empirical evidence on the existence of structural and functional connections between LS-VOT and other brain parts. Secondly, we might wonder what kind of theory would describe the role of LS-VOT in such distributed network/s.

The functional and structural connectivity may constrain the extent of potential networks and therefore shed light on the cognitive characteristics of LS-VOT (e.g., Pessoa, 2014; Chen et al., 2019). The local or sparse connections with other brain areas will restrict the role of the region of interest. In the case of LS-VOT, a number of connections both structural and functional connections were found, with many distant regions within the language network, which exist even before the reading acquisition. According to the *biased connectivity hypothesis* (Dehaene and Dehaene-Lambertz, 2016; Moulton et al., 2019) the location of LS-VOT is directly determined by the structural connections to other cortical language areas.

The connectivity of LS-VOT with the language network was studied from the earliest stages of development. Li et al. (2020) studied newborns within 1 week after birth. They examined the resting state activity that showed significant connectivity between anterior LS-VOT and frontal and temporal language networks. Saygin et al. (2016) scanned 5-year-olds before they learned to read. At the age of eight children were tested again with tasks that determined the functional localization of VWFA. The VWFA area even before the reading acquisition had white matter (anatomical fiber tracts) connections with speech and language processing networks. Moulton et al. (2019) showed that microstructural changes in the pathway between LS-VOT and the left inferior parietal lobule (IPL) correlated with the reading level in the first year of reading instruction. Stevens et al. (2017) asked if the functional connectivity between VWFA and the language system is specifically tuned to processing words vs. other visual stimuli like namable pictures. They showed that resting-state connectivity of LS-VOT with the left posterior STG is preferential compared to other visual stimuli. Also, Bouhali et al. (2014) compared VWFA with the fusiform face area (FFA) and proved privileged connectivity for VWFA to left perisylvian superior temporal and inferior frontal areas (but for a domain-general view see: Vogel et al., 2013).

The interesting question in this field of research is how to explain the role of the preferential connectivity between some parts of VOT and the language network even before children start to read. The answer to this question might advance our knowledge of the VOT role in language processing in general. The simplest hypothesis is that connections between VOT and language systems are necessary for naming visual stimuli. Stevens et al. (2017) showed that in the semantic classification task (deciding whether the stimuli were man-made or natural objects), the connections between the left posterior STG and LS-VOT predicted only performance on word stimuli compared to picture stimuli. The authors proposed that while structural connectivity does not drive specialization, the experience-driven “coupling among regions critical for performance during skill acquisition” (p. 5296) leads to higher connectivity of those regions over time. However, longitudinal studies are needed since the preferential connectivity of LS-VOT with language networks in adults might be a result of reading training not a cause of LS-VOT engagement in reading.

The experience-driven hypothesis of the LS-VOT specialization for language is supported by the studies on the blind population. As mentioned before in the early and congenitally blind LS-VOT is engaged in speech processing on a higher level (e.g., in tasks demanding syntactic processing) than it is observed in the sighted population. According to the pluripotent cortex hypothesis (Bedny, 2017) such activity may be an effect of similar structural connectivity of the VOT with other brain regions but changed experience, as no visual input enters this region in the blind. The connections with the language processing areas in this context may get strengthened (Abboud and Cohen, 2019; Kanjlia et al., 2021) and lead to increased sensitivity to language early on in the development of blind individuals. The LS-VOT would thus be a part of distributed, modality-independent language network (Dziȩgiel-Fivet et al., 2021) even before reading acquisition and become specialized for Braille reading only afterward in a similar manner in which frontal and temporal areas become engaged in the visual reading. This hypothesis was further supported by the observation that in the blinds LS-VOT lacks the posterior-to-anterior organization gradient that is observed in the sighted (Tian et al., 2022). Instead, such a gradient can be observed in the modality-specific posterior-parietal cortex in the blind.

As for the comprehensive theory of the role of LS-VOT in a distributed system the most known Interactive Account was given by Price and Devlin (2011). According to their proposal, LS-VOT is not responsible for encoding orthographic or visual information but serves as an “interface” between sensory input and top-down predictions modulated by attentional and task requirements. In default “sensory inputs” are visual properties of words and “top-down predictions” correspond to the non-visual features of stimuli like phonological or semantic information. However, the authors see linguistic processing as a distributed system where it is difficult to dissociate the parts of cognitive functions that are implemented in a given region of the network. According to that view, “orthographic representations emerge from the interaction of backward and forward influences” (Price and Devlin, 2011, paragraph 3). The authors use a “prediction error” concept: it results from the top-down influence of linguistic information. Greater prediction error (mismatch between visual input and top-down predictions) evokes higher activity of the LS-VOT. This, according to the authors, explains why the LS-VOT activates more strongly for pseudowords than words: real words cause the lowest prediction error. This is an alternative explanation to the “Familiarity effect” (Kronbichler et al., 2004; discussed in paragraph 3) or “Rapid Adaptation effect” (Glezer et al., 2009, 2016) where the LS-VOT is less active on known stimuli because of the adaptation processes. However, the context (e.g., adding attentional demands) might change the activity pattern of the LS-VOT. In consequence, its activity should be disturbed and overall lower in reading deficit because linguistic top-down predictions are less precise. The developmental stage also matters: LS-VOT activity should be highest at the beginning of learning to read because top-down predictions are only starting to be formed. The preliminary empirical evidence was provided, e.g., by Wang et al. (2022): based on graph analysis results the authors claimed that the LS-VOT serves as an “interface” between visual and higher-order systems. In some tasks (sentence comprehension) LS-VOT becomes a connector in the auditory-sensorimotor system whereas in speech processing in noise when the signal is severely degraded LS-VOT serves as a peripheral part of a visual system.

White et al. (2023) also showed a task-dependent modulation of LS-VOT. In the case of processing written words, the task to recognize them (vs. ignoring them) resulted in an increased level of LS-VOT activity compared to processing symbol strings. The task-dependent modulation for words was associated with higher functional connectivity with language regions and was only visible in a part of LS-VOT that corresponds to VWFA but not in the overall visual cortex. It shows that the LS-VOT region is modulated by the need for reading words but not processing other visual stimuli.

Another proposal suggesting that the LS-VOT is part of a distributed neural system was formulated by Chen et al. (2019). The authors showed results in favor of a multiplex model of LS-VOT. They examined structural connectivity between LS-VOT and *a priori* defined regions of the language and attention networks. LS-VOT showed an even stronger connection with the attention network (frontoparietal nodes) than with the language network. Also, the double dissociation in brain-behavior relation was discovered: the level of structural connectivity between the LS-VOT and nodes within the language network was found to be a predictor of individual differences in reading and language abilities, but not attention. Still the degree of structural connectivity between the LS-VOT and nodes in the dorsal frontoparietal attentional network was associated with visual attention abilities, but not with reading and language abilities. The results are particularly interesting in light of the discussion on the LS-VOT involvement in speech processing because most of the phonological tasks evoking activity of LS-VOT were attention-based (e.g., phoneme deletion or rhyme matching) compared to passive listening of speech. However, the multiplex model assumes interaction between attention and language in the left VOT – not necessarily two distinct roles of the left VOT. In general, thanks to the local connections with the visual system and distant relations with the attentional and language systems the left VOT is an “attentional spotlight” on word-like stimuli that are later processed in a language network. Woodhead et al. (2014) studied the role of the left IFG in word recognition. They found that the left IFG is sensitive to words and provides top-down feedback to the VOT, aiding in efficient word identification. This feedback occurs in the early stages of processing. In line with this highly interactive view, Ellenblum et al. (2019) showed that the orthographic network is not as coherent as other well-described networks (like DMN). The authors explained that the orthographic process requires highly specialized cross-domain and cross-modality interactions and many already complex cognitive functions therefore they point to the “high-level integrative” role of the LS-VOT in reading and spelling.

## 6. LS-VOT acquires its function in the development

Based on early connectivity studies of structural and functional connections of LS-VOT in childhood (as in e.g., Saygin et al., 2016) different developmental paths of LS-VOT involvement in reading and language processing might be formulated. The canonical approach comes from Dehaene, Cohen, and colleagues (e.g., Dehaene and Cohen, 2007) proposal that we may call here “the orthographic hypothesis.” In this view, the development of orthographic sensitivity in LS-VOT, which is primarily responsible for complex visual stimuli processing, is driven by literacy development. Then possibly automatic orthographic co-activations grow during phonology processing, which makes LS-VOT sensitive to speech processing even without the visual stimuli present.

In detail, the original neuronal recycling hypothesis claims that when we learn to read, our brain repurposes certain areas of the left LS-VOT previously used for recognizing other things like faces or objects. The process of neuronal recycling would involve a competition in which the group of neurons used in print processing “invades” the regions of the LS-VOT and takes them over for reading (Dehaene and Cohen, 2007). The hypothesis has been prominent since it was proposed and referenced in studies on both adults (Dehaene et al., 2010; Pegado et al., 2014) and children (Monzalvo and Dehaene-Lambertz, 2013). Studies comparing literate and illiterate adults have found that LS-VOT regions involved in reading also respond to other visual stimuli like objects, faces, and checkerboards. As reading ability improved, responses to faces decreased slightly in the left side of the brain and increased significantly in the right side (Dehaene et al., 2010; Pegado et al., 2014). Similarly in children, right lateralization of the activation to faces in the VOT increased with reading performance (Monzalvo et al., 2012). More recently, also a “weaker” or “revised” version of the hypothesis has been proposed, based on the longitudinal data (Dehaene-Lambertz et al., 2018). The revised hypothesis suggests that the brain has dedicated areas for processing different visual categories, which expand as we grow. With reading instruction, the LS-VOT responds to written words at initially weakly specialized sites, and without altering their (weak) preexisting responsivity to other visual inputs, such as faces. This view was also supported by recent studies that reported co-existence of growing word sensitivity and other visual categories representations (Nordt et al., 2021; Feng et al., 2022; Chyl et al., 2023).

Nonetheless, the relationship between the emergence of different visual categories and competition in the LS-VOT is currently far from being a consensus, and the hypothesis has been critically discussed. New research has found that literacy can actually enhance the similarity between representations of text and faces, without reducing the response to other, non-linguistic visual categories (Hervais-Adelman et al., 2019; Van Paridon et al., 2021). In fact, the recent critical review (Rossion and Lochy, 2022) found little evidence to support the idea that reading ability affects the way the brain processes faces. Instead, the review argued that right-lateralized neural activity for face recognition emerges early in life, and is not modulated by literacy level. Therefore, it is hard to say what is actually “recycled” if word selectivity invades cortical regions that are not selective (or weakly selective) to faces or other categories, as the “revised” version of the hypothesis postulates. Hence, the authors suggest abandoning the misleading “recycling” terminology altogether, not just “revising” it (Rossion and Lochy, 2022). They propose the term “neural competition” in which sensory inputs sharing functional characteristics (e.g., faces and words) compete for the same population of neurons within the left VOT. Also, Vogel et al. (2012)^2014^ studies on adults showed that the acquisition of lexical representations does not necessarily replace the ability of LS-VOT for visual analysis. Instead this region remains crucial for reading thanks to its sensitivity to visual characteristics of print and its involvement in statistical learning that enables fluent processing of real words. However, the same region might be useful for processing other types of information.

The alternative pathway of LS-VOT functional development might be called “the phonology to orthography hypothesis.” This alternative was formulated by Conant et al. (2020) who claimed that areas within VOT are involved in speech-face circuitry because of the proximity to FFA. After reading acquisition, some parts of LS-VOT that were involved in processing speech become responsible for audiovisual integration between phonology and orthography and later LS-VOT starts to be responsible for processing unimodal orthographical forms. This alternative explains what might be the evolutionary relevant function of the connections between some parts of the VOT and the language networks before reading acquisition. If this is the case, the preferential activity for speech sounds in LS-VOT should be observed already in small children before schooling. So far, there is evidence of LS-VOT involvement in phonological processing at the beginning of reading acquisition but not in true pre-readers without any experience with print or letter knowledge. For example, Dȩbska et al. (2016) showed stronger LS-VOT involvement in 6–7 years old Polish children in an auditory rhyming task in a group without the familial risk for dyslexia compared to the risk group. Similarly, Wang J. et al. (2018) showed that activity of the posterior left VOT correlated with a reading level in an auditory onset comparison task in 5–6 years old beginning readers in English. Also, the activity of the posterior left VOT was related to efficiency in a phonological in-scanner task. However, Dehaene et al., (2010, 2015) claimed that LS-VOT is not sensitive to speech or phonology in illiterate adults. Therefore more evidence on preliterate children is needed to prove the preferential engagement of LS-VOT in speech processing.

Another promising but less studied explanation is “the growing multimodality hypothesis.” First, the sensitivity for orthography in LS-VOT might be explained to be a consequence of a multimodal interaction during establishing letter–speech sound correspondences (Brem et al., 2010). This recurring co-activation in the reading process might lead to establishing an ortho-phonological representation based on connectivity with a speech processing network in the left STG. In this view, there are no “pure” orthographic representations that are not related to phonology – at least in a typical development of reading. Analogically the constant co-activation may lead to the restructuring of primary phonological areas like the left STG in a way that this region becomes sensitive to strictly orthographic effects like consistency in spelling (as proposed by Pattamadilok et al., 2010).

## 7. Conclusion

The research described in this article provides evidence that the term “Visual Word Form Area” is insufficient to describe the cognitive role of the language specific - VOT (LS-VOT) area. Different phonological, orthographic, and semantic functions of the LS-VOT were found that turned out to be independent of modality. The most promising approach for defining a general cognitive function of the LS-VOT seems to be focusing on its preferential structural and functional connectivity with higher-level language and attention networks. In this view, the LS-VOT may be described as a task-driven “interactive gateway” that connects certain modality-independent stimuli with their associated orthographic, semantic, and phonological properties processed within the whole network. The activity of the LS-VOT is not limited to a specific script or orthography system but extends to all symbolic communication-relevant stimuli that convey meaning in a repeatable manner.

Overall the ventral visual pathway may play a more major role as an interactive hub connecting modality-independent experience with brain regions specialized for different functions. The shift toward a domain-general perspective in understanding the functions of the ventral stream is visible in studies on processing numbers (Chen and Verguts, 2012, 2017; Grotheer et al., 2019). Specialization in the ventral “visual” stream can develop regardless of sensory modality or visual experience (Abboud et al., 2015; Grotheer et al., 2016). This phenomenon occurs due to unique patterns of connectivity (Grotheer et al., 2019). Similar findings have been observed in studies on action-object recognition, categorization processes (Grill-Spector et al., 2001; Mahon et al., 2007, 2009) and semantic representations (Chen and Mirman, 2015). A crucial area of focus for future research is to determine whether the functional connectivity between the ventral stream and other structures beyond it serves as a fundamental organizing principle that leads to category-specific processing in the human visual system.

## Author contributions

AD contributed to the conceptualization and wrote the first draft of the manuscript. MW, KC, and GD-F wrote sections of the manuscript. KC prepared the figures. KJ revised the manuscript. All authors contributed to manuscript revision, read, and approved the submitted version.
